# Color Image Segmentation Based on Different Color Space Models Using Automatic GrabCut

**DOI:** 10.1155/2014/126025

**Published:** 2014-08-31

**Authors:** Dina Khattab, Hala Mousher Ebied, Ashraf Saad Hussein, Mohamed Fahmy Tolba

**Affiliations:** ^1^Faculty of Computer and Information Sciences, Ain Shams University, Cairo 11566, Egypt; ^2^Faculty of Computer Studies, Arab Open University-Headquarters, 13033 Al-Safat, Kuwait

## Abstract

This paper presents a comparative study using different color spaces to evaluate the performance of color image segmentation using the automatic GrabCut technique. GrabCut is considered as one of the semiautomatic image segmentation techniques, since it requires user interaction for the initialization of the segmentation process. The automation of the GrabCut technique is proposed as a modification of the original semiautomatic one in order to eliminate the user interaction. The automatic GrabCut utilizes the unsupervised Orchard and Bouman clustering technique for the initialization phase. Comparisons with the original GrabCut show the efficiency of the proposed automatic technique in terms of segmentation, quality, and accuracy. As no explicit color space is recommended for every segmentation problem, automatic GrabCut is applied with *RGB*, *HSV*, *CMY*, *XYZ*, and *YUV* color spaces. The comparative study and experimental results using different color images show that *RGB* color space is the best color space representation for the set of the images used.

## 1. Introduction

The process of partitioning a digital image into multiple segments is defined as image segmentation. Segmentation aims to divide an image into regions that can be more representative and easier to analyze. Such regions may correspond to individual surfaces, objects, or natural parts of objects. Typically image segmentation is the process used to locate objects and boundaries (e.g., lines or curves) in images [1]. Furthermore, it can be defined as the process of labeling every pixel in an image, where all pixels having the same label share certain visual characteristics [2]. Usually segmentation uses local information in the digital image to compute the best segmentation, such as color information used to create histograms or information indicating edges, boundaries, or texture information [3].

Color image segmentation that is based on the color feature of image pixels assumes that homogeneous colors in the image correspond to separate clusters and hence meaningful objects in the image. In other words, each cluster defines a class of pixels that share similar color properties. As the segmentation results depend on the used color space, there is no single color space that can provide acceptable results for all kinds of images. For this reason, many authors tried to determine the color space that will suit their specific color image segmentation problem [4]. In this work, a segmentation of color images is tested with different classical color spaces, *RGB*, *CMY*, *XYZ*, *YUV*, and *HSV*, to select the best color space for the considered kind of images.

The segmentation process is based on the GrabCut segmentation technique [5], which is considered as one of the powerful state-of-the-art techniques for the problem of color image segmentation. The iterative energy minimization scheme of the GrabCut is based on the powerful optimization of the Graph Cut technique [6] which allows for the generation of the global optimal segmentation. In addition, Graph Cut can be easily well extended to the problem of N-D images. Furthermore, the cost energy function of the Graph Cut minimization process allows it to be defined in terms of different image features such as color, region, boundary, or any mixture of image features. This flexibility provides wide potential for the use of GrabCut in different applications. On the other hand, GrabCut is considered as a bilabel segmentation technique, where images can be segmented into two background and foreground regions only. Initial user intervention is required in order to specify an object of interest to be segmented out of the image, considering all the remaining image pixels as one background region. This classifies the GrabCut as a semiautomatic segmentation technique and turns the quality of the initialization and hence the segmentation performance, sensitive to the user selection. In other words, poor GrabCut initialization may lead to bad final segmentation accuracy which might require extra user interactions with the segmentation results for fine tuning [5].

In this work, a modified GrabCut is proposed as an automatic segmentation technique, which can segment the image into its natural objects without any need for the initial user intervention. Automation of GrabCut is applied using Orchard and Bouman clustering [7] as an unsupervised clustering technique. The selection of the Orchard and Bouman clustering is based on the empirical comparison results carried out in the work of [8]. The paper exploits the use of some evaluation criteria to evaluate the discriminating power of the automatic GrabCut with the different color spaces. The remainder of the paper is organized as follows.  Section 2 provides a basic background on segmentation based-color space models, image segmentation using GrabCut, and unsupervised clustering techniques.  Section 3 explains the different color space models.  Section 4 illustrates the Orchard and Bouman clustering. The original GrabCut technique and details of its modification are explained in Section 5. Experimental results are presented in Section 6, while the conclusion and future work are presented in Section 7.

## 2. Related Work

As no common opinion has emerged about which is the best choice for color space based image segmentation, some research work tried to identify the best color space for a specific task. Several works [9, 10] show that different color spaces are useful for the problem of color image segmentation. Jurio et al. [11] have carried out a comparative study between different color spaces in cluster based image segmentation using two similar clustering algorithms. Their study involved the test of four color spaces, *RGB*, *HSV*, *CMY*, and *YUV*, in order to identify the best color representation. They obtained their best results in most cases using *CMY* color space, while *HSV* also provided good results. Busin et al. [4] proposed a method to automatically select a specific color space among classical color spaces. This selection was done according to an evaluation criterion based on a spectral color analysis. This criterion evaluates the quality of the segmentation in each space and selects the best one, which preserves its own specific properties. A study of the ten most common color spaces for skin color detection was presented in [12]. They concluded that *HSV* is the best color space to detect skin in an image. Another study that was applied for the classification of pizza toppings [13] proved that the polynomial SVM classifier combined with *HSV* color space is the best approach among five different color spaces. Based on a comparative study between the *RGB* and *HSV* models, Ruiz-Ruiz et al. [14] declared that the best accuracy was achieved with *HSV* representation in order to achieve real time processing in real farm fields for crop segmentation.

GrabCut is considered one of the powerful techniques used for color image segmentation. It has been applied to different segmentation problems such as human body segmentation [15–17], video segmentation [18], semantic segmentation [19], and volume segmentation [20]. In [17], an automatic extraction of the human body from color images was developed by Hu. The iterated GrabCut technique was used to dynamically update a trimap contour, which was initialized from the results of a scanning detector used for detecting faces from images. The research has some drawbacks as the process goes through many steps and iterations, in addition to being constrained to human poses with frontal side faces. A fully automatic Spatio-Temporal GrabCut human segmentation methodology was proposed by Hernandez et al. [16]. They developed methodology that takes the benefits of the combination of tracking and segmentation. Instead of the initial user intervention to initialize the GrabCut algorithm, a set of seeds defined by face detection and a skin color model are used for initialization. Another approach to segment humans from cluttered images was proposed by Gulshan et al. in [15]. They utilized the local color model based GrabCut for automatic segmentation. This GrabCut local color model was used to refine the crude human segmentations they obtained. In video segmentation, Corrigan et al. [18] extended GrabCut for more robust video object segmentation. They extended the Gaussian mixture model (GMM) of the GrabCut algorithm, so that the color space was complemented with the derivative in time of the pixel's intensities in order to include temporal information in the segmentation optimization process. Göring et al. [19] integrated GrabCut into a semantic segmentation framework by labeling objects in a given image. Most recently, Ramírez et al. [20] proposed a fully parallelized scheme using GrabCut for 3D segmentation that has been adopted to run on GPU. The scheme aims at producing efficient segmentation results for the case of volume meshes, in addition to reducing the computational time.

Clustering [21], the unsupervised classification of patterns into groups, is one of the most important tasks in exploratory data analysis [22]. It has a long and rich history in a variety of scientific disciplines including anthropology, biology, medicine, psychology, statistics, mathematics, engineering, and computer science. Clustering in image segmentations [2, 23, 24] is defined as the process of identifying groups of similar image primitives. Unsupervised clustering techniques [25] are content based clustering, where content refers to shapes, textures, or any other information that can be inherited from the image itself.

In the cases of bilabel segmentation, good separation between foreground and background is required. This can be implemented through finding clusters with a low variance, since this makes the cluster easier to separate from the others. The selection of the Orchard and Bouman clustering technique [7] is guided by Ruzon and Tomasi [26] and Chaung et al. [27] in order to get tight and well separated clusters. They have worked on solving the problem of image matting that is required for image compositing. In their approach, Orchard and Bouman binary split algorithm has been used for partitioning the unknown region colors into several clusters, in order to generate a color distribution for the unknown region to be estimated. According to a comparative study in [8], the Orchard and Bouman clustering outperformed other unsupervised clustering techniques including self-organizing maps (SOFM) and fuzzy C-means (FCM) for the automation of the GrabCut in terms of improving the segmentation accuracy.

## 3. Color Space Models

The most widely used color space is the *RGB* color space, where a color point in the space is characterized by three color components of the corresponding pixel which are red (*R*), green (*G*), and blue (*B*). However since there exist a lot of color spaces, it is useful to classify them into fewer categories with respect to their definitions and properties. Vandenbroucke [28] proposed the classification of the color spaces into the following categories.

(i) The primary spaces which are based on the theory that assumes it is possible to match any color by mixing an appropriate amount of the three primary colors: the primary spaces are the real *RGB*, the subtractive *CMY*, and the imaginary *XYZ* primary spaces. The conversion from *RGB* to *CMY* is
  (1) $$\begin{array}{c} C^{\prime }=1-R\;\;C=\min\left(1,\max\left(0,C^{\prime }-K^{\prime }\right)\right)\\ M^{\prime }=1-G\;\;M=\min\left(1,\max\left(0,M^{\prime }-K^{\prime }\right)\right)\\ Y^{\prime }=\;1-B\;\;Y=\min\left(1,\max\left(0,Y^{\prime }-K^{\prime }\right)\right)\\ K^{\prime }=\min\left(C^{\prime },M^{\prime },Y^{\prime }\right) \end{array}$$

and the conversion from *RGB* to *XYZ* is

(2) $$\begin{array}{c} \left[\begin{array}{c} X\\ Y\\ Z \end{array}\right]=\left[\begin{array}{ccc} 0.412453 & 0.357580\; & 0.180423\;\\ 0.212671 & 0.715160 & 0.072169\\ 0.019334 & 0.119193 & 0.950227 \end{array}\right]\left[\begin{array}{c} R\\ G\\ B \end{array}\right]. \end{array}$$

(i) The luminance-chrominance spaces, which are computed of one color component that represents the luminance and two color components that represent the chrominance: the *YUV* color space is an example of the luminance-chrominance spaces. The conversion from *RGB* to *YUV* is
  (3) $$\begin{array}{c} \left[\begin{array}{c} Y\\ U\\ V \end{array}\right]=\left[\begin{array}{ccc} 0.2989 & 0.5866 & 0.1145\\ -0.147 & -0.289 & 0.436\\ 0.615 & -0.515 & -0.100 \end{array}\right]\left[\begin{array}{c} R\\ G\\ B \end{array}\right]. \end{array}$$
(ii) The perceptual spaces that try to quantify the subjective human color perception by means of three measures, intensity, hue, and saturation: the *HSV* is an example of the perceptual color space. The conversion from *RGB* to *HSV* is
  (4) $$\begin{array}{c} H=\{\begin{array}{cc} 0, & \text{if}\;\mathrm{Max}=\text{Min }\\ \left(60^{{}^{\circ}}\times \frac{G-B}{\mathrm{Max}-\mathrm{Min}}+360^{{}^{\circ}}\right) & \\ \;\times \mathrm{mod}360^{{}^{\circ}}, & \text{if}\;\mathrm{Max}=R\\ 60^{{}^{\circ}}\times \frac{B-R}{\mathrm{Max}-\mathrm{Min}}+120^{{}^{\circ}}, & \text{if}\;\mathrm{Max}=G\\ 60^{{}^{\circ}}\times \frac{R-G}{\mathrm{Max}-\mathrm{Min}}+240^{{}^{\circ}}, & \text{if}\;\mathrm{Max}=B \end{array}\\ S=\{\begin{array}{cc} 0, & \text{if}\;\max=0\\ \frac{\mathrm{Max}-\mathrm{Min}}{\mathrm{Max}} & \text{otherwise} \end{array}\\ V=\mathrm{Max}. \end{array}$$

## 4. Orchard and Bouman Clustering Technique

Orchard and Bouman [7] is a color quantization clustering technique that uses the eigenvector of the color covariance matrix to determine good cluster splits. The algorithm starts with all the pixels in a single cluster. The cluster is then split into two using a function of eigenvector of the covariance matrix as the split point. Then it uses the eigenvalues of the covariance matrices to choose which of the resulting clusters is candidate for the next splitting. This procedure is repeated until the desired number of clusters is achieved. It is an optimal solution for large clusters with Gaussian distributions.

For example, consider *C*_1_ as a set of pixels, in order to divide it into *K* clusters:

1. calculate *μ*_1_, the mean of *C*_1_, and Σ_1_, the covariance matrix of *C*_1_,
2. for *i* = 2 to *K* do the following:
  - find the set *C*_*n*_ which has the largest eigenvalue and store the associated eigenvector *e*_*n*_,
  - split *C*_*n*_ into two sets, *C*_*i*_ = {*x* ∈ *C*_*n*_ : *e*_*n*_^*T*^ *z*_*n*_ ≤ *e*_*n*_^*T*^ *μ*_*n*_} and *C*_n_* = *C*_*n*_ − *C*_*i*_,
  - compute *μ*_n_*, Σ_n_*, *μ*_*i*_, and Σ_*i*_.

This results in *K* pixel clusters.

## 5. Image Segmentation Using GrabCut

Image segmentation is simply the process of separating an image into foreground and background parts. Graph Cut technique [6] was considered as an effective way for the segmentation of monochrome images, which is based on the Min-Cut/Max-Flow algorithm [29]. GrabCut [5] is a powerful extension of the Graph Cut algorithm to segment color images iteratively and to simplify the user interaction needed for a given quality of the segmentation results.  Section 5.1 explains the original semiautomatic GrabCut algorithm as developed by Rother et al. in [5], while its modification for automatic segmentation is presented in Section 5.2.

### 5.1. Original Semiautomatic GrabCut

The GrabCut algorithm learns the color distributions of the foreground and background by giving each pixel a probability to belong to a cluster of other pixels. It can be explained as follows: given a color image I, let us consider the *z* = (*z*_1_,…, *z*_*n*_,…, *z*_*N*_) of *N* pixels, where *z*_*i*_ = (*C*_1*i*_, *C*_2*i*_, *C*_3*i*_), *i* ∈ [1,…, *N*], and *C*_*j*_ is the *j*th color component in the used color space. The segmentation is defined as an array *α* = (*α*_1_,…, *α*_*N*_), *α*_*i*_ ∈ {0,1}, assigning a label to each pixel of the image, indicating if it belongs to the background or the foreground. The GrabCut algorithm consists mainly of two basic steps: initialization and iterative minimization. The details of both steps are explained in the following subsections.

#### 5.1.1. GrabCut Initialization

**Figure 1:**
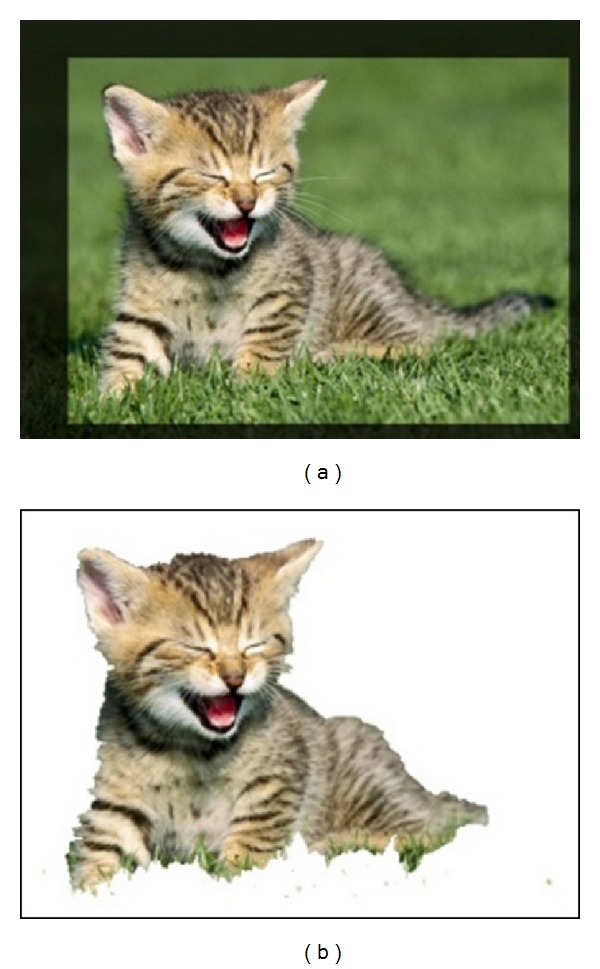
Example of GrabCut segmentation. (a) GrabCut allows the user to drag a rectangle around the object of interest to be segmented. (b) The segmented object.

The novelty of the GrabCut technique is in the “incomplete labeling” which allows a reduced degree of user interaction. The user interaction consists simply of specifying only the background pixels by dragging a rectangle around the desired foreground object (Figure 1). The process of GrabCut initialization works as follows.

**Step 1 .**

A trimap *T* = {TB, TU, TF} is initialized in a semiautomatic way. The two regions TB and TU contain the initial background and uncertain pixels, respectively, while TF = *Ø*. The initial TB is determined as the pixels around the outside of the marked rectangle. Pixels belonging to TB are considered as a fixed background, whereas those belonging to TU will be labeled by the algorithm.

**Step 2 .**

An initial image segmentation *α* = (*α*_1_,…, *α*_*i*_,…, *α*_*N*_), *α*_*i*_ ∈ {0, 1}, is created, where all unknown pixels are tentatively placed in the foreground class (*α*_*i*_ = 1 for *i* ∈ TU) and all known background pixels are placed in the background class (*α*_*i*_ = 0 for *i* ∈ TB).

**Step 3 .**

Two full covariance Gaussian mixture models (GMMs) are defined, each consisting of *K* = 5 components, one for background pixels (*α*_*i*_ = 0) and the other one for foreground (initially unknown) pixels (*α*_*i*_ = 1). The *K* components of both GMMs are initialized from the foreground and background classes using the Orchard and Bouman clustering technique.

#### 5.1.2. GrabCut Iterative Energy Minimization

**T**he final segmentation is performed using the iterative minimization algorithm of the Graph Cut [6] in the following steps.

**Step 4 .**

Each pixel in the foreground class is assigned to the most likely Gaussian component in the foreground GMM. Similarly, each pixel in the background is assigned to the most likely background Gaussian component.

**Step 5 .**

The GMMs are thrown away and new GMMs are learned from the pixel sets created in the previous set.

**Step 6 .**

A graph is built and Graph Cut is run to find a new foreground and background classification of pixels.

**Step 7 .**

Steps 4–6 are repeated until the classification converges.

This has the advantage of allowing the automatic refinement of the opacities *α*, as newly labeled pixels from the TU region of the initial trimap are used to refine the color of the GMM.

### 5.2. Proposed Automatic GrabCut

Although the incomplete user labeling of GrabCut reduces the user interaction substantially, it is still a requirement in order to initiate the segmentation process. This identifies GrabCut as a semiautomatic/supervised segmentation algorithm. In order to allow the image to be segmented into proper segments without any user guidance, this requires replacing the semiautomatic/supervised step of GrabCut initialization with a totally automatic/unsupervised one.

In this paper, the Orchard and Bouman [7] is proposed to be used as an image clustering technique to automatically set the initial trimap *T* and the initial segmentation (Section 5.1, Steps 1 and 2). The distinction between the trimap and the segmentation formalizes the separation between the region of interest to be segmented and the final segmentation derived by the GrabCut algorithm. In the automatic technique, Steps 1 and 2 of the GrabCut initialization process will be modified as follows.

**Step 1 .**

While the original GrabCut constructs a trimap *T* of two regions, TB and TU, as a fixed background and unknown regions, respectively, the proposed automatic technique considers the whole image as one unknown region TU, where TU = {*z*_*i*_ ∈ {*z*_1_,…, *z*_*n*_,…, *z*_*N*_}}, *i* ∈ [1,…, *N*]. This means that no fixed foreground or background regions are known and all image pixels will be involved in the minimization process to be labeled by the algorithm.

**Step 2 .**

The image is initially separated into two foreground TF and background TB regions, using the Orchard and Bouman clustering technique. During this step, a new GMM is introduced, which consists of only two components (*K* = 2): one component for the background pixels (*α*_*i*_ = 0) and the other for the foreground pixels (*α*_*i*_ = 1). The Orchard and Bouman clustering technique is then applied and repeated until reaching the number of components (*K* = 2) in the GMM, resulting in separating the image exactly into two clusters.

**Step 3 .**

The colors of image pixels belonging to each cluster (foreground and background clusters) generated from the previous step are then used to initialize another two full covariance Gaussian mixture models (GMMs) with (*K* = 5).

*Steps 4–7*. The learning portion of the algorithm runs exactly as the original GrabCut (Section 5.1, Steps 4–7).

## 6. Results and Discussions

**Figure 2:**
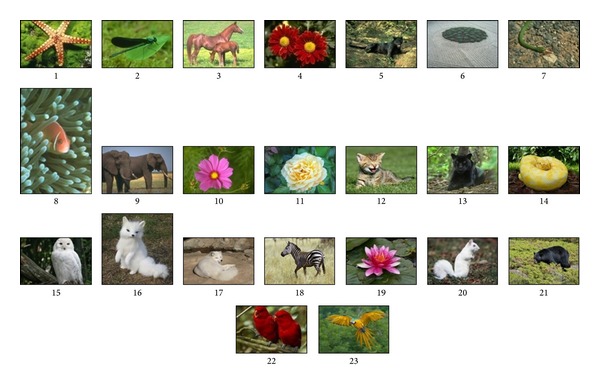
The dataset of images.

The automatic GrabCut technique was experimentally tested using a dataset of 23 different images, as shown in Figure 2. According to literature, many recent works in the fields of cluster based image segmentation and automatic image segmentation are conducting their experiments on fewer numbers of images such as [9, 11, 30, 31]. They are using a dataset of 8, 4, 4, and 15 images, respectively. In this work using a dataset of 23 images can be considered a reasonable number of test cases. This dataset is collected partially from the Berkeley segmentation dataset [32] and from publically available images [33] in a way that matches certain criteria. These criteria consider a special fitting into two class segmentations, including having mainly one object (as a foreground) and a well separation between the foreground and background color regions.

**Figure 3:**
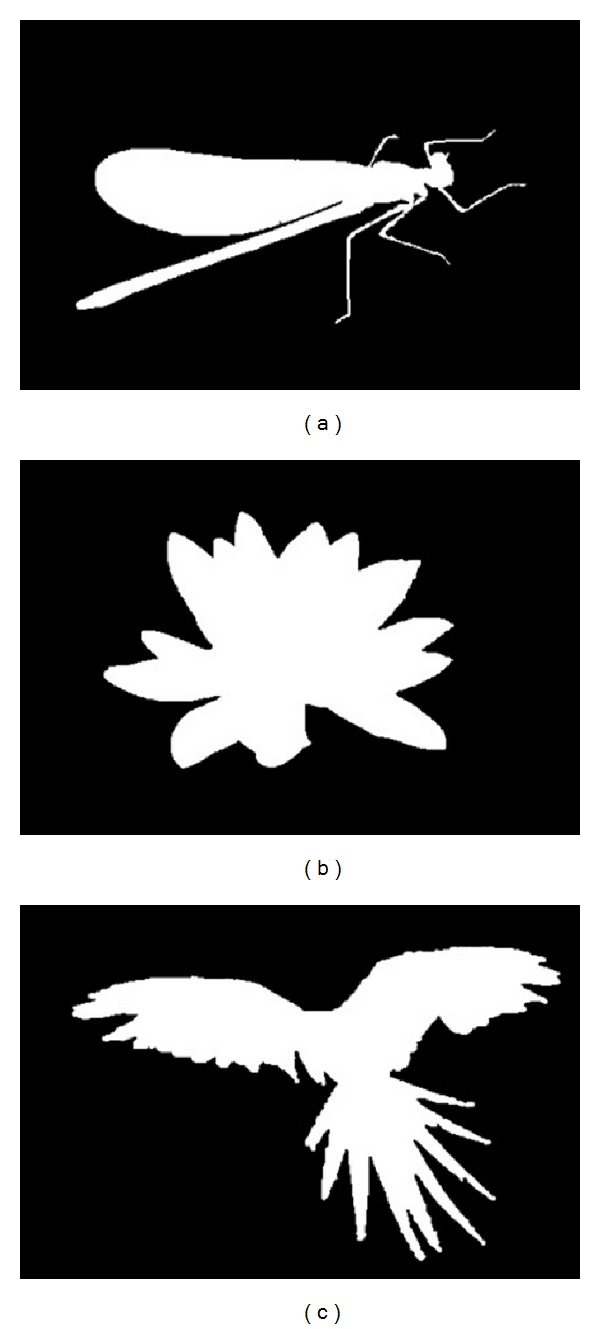
Samples of the manual binary ground truths generated.

For evaluation, it was noticed that no binary segmentations exist as part of the human segmentations included in the Berkeley segmentation dataset [32]. For this reason, the ground truth data for our selected dataset is manually generated using standard image processing tools (Adobe Photoshop).  Figure 3 displays samples of the manual binary ground truths generated. The error rate and the overlap score rate are used as two evaluation metrics. The error rate is calculated as the fraction of pixels with wrong segmentations (compared to ground truth) divided by the total number of pixels in the image. The overlap score rate is given by *y*_1_∩*y*_2_/*y*_1_ ∪ *y*_2_, where *y*_1_ and *y*_2_ are any two binary segmentations.

**Figure 4:**
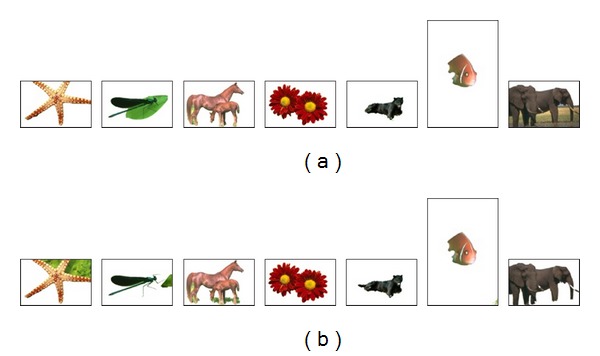
Visual comparison of the segmentation results of (a) original semiautomatic GrabCut and (b) automatic GrabCut initialized using Orchard and Bouman.

In the first experiment, automatic GrabCut, which is initialized using Orchard and Bouman, is applied and compared to the original GrabCut algorithm.  Figure 4 shows sample visual results for the segmentation using (*K* = 5) components for GMMs as recommended by Rother et al. [5].  Table 1 shows the quantitative comparison between the original and modified GrabCut for the whole dataset as presented in Figure 2. As shown in Table 1, the automatic GrabCut using Orchard and Bouman clustering outperforms the original one in terms of minimizing the error and improving the segmentation accuracy. The average error rate is 3.64% for the automatic GrabCut compared to 4.28% for the original GrabCut technique. The overall performance looks better in terms of the standard deviation (SD) which exhibits 3.61% for the automatic GrabCut compared to 5.5% for the original GrabCut.

Some cases with bad segmentation error using the original GrabCut can be noticed in Table 1 (images 1 and 9). This explains one main drawback of the original GrabCut initialization, which makes the segmentation results sensitive to the user selection of the area of interest to be segmented. This occurs when other objects, which are out of interest, may be considered as part of the foreground by being located within the area of the dragged rectangular boundary around the object of interest. The segmentation results of these two images are visually illustrated in Figure 4(a). It can be noticed how a large portion of the leaf appears in the final segmentation of the insect image. The same problem occurred when considering the land as part of the foreground area with the elephant image. The quantitative comparisons of the error rates generated for these two images in Table 1 and visual comparisons in Figure 4 illustrate the efficiency of the automatic GrabCut in handling such a problem. The efficiency of the automatic GrabCut is provoked by preventing any hard constraints to be specified during initialization either for foreground or background (Section 5.2, Step 1).

**Figure 5:**
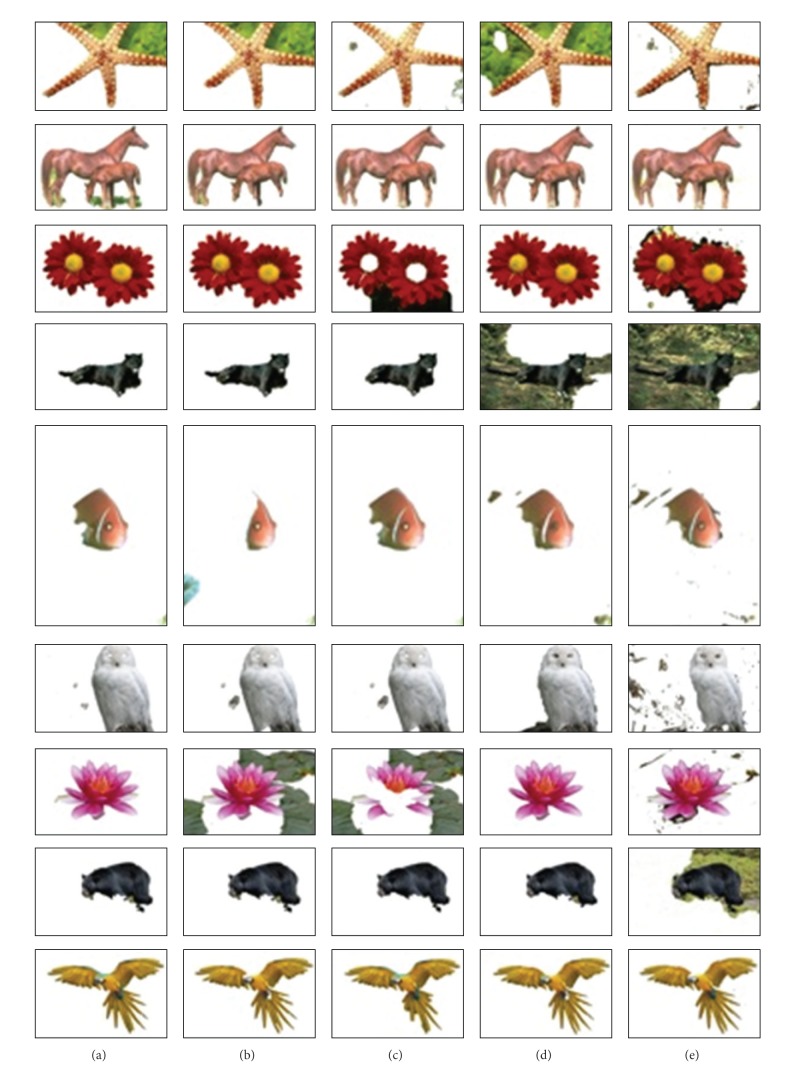
Visual comparison of segmentation results for automatic GrabCut applied in the (a) *RGB*, (b) *YUV*, (c) *XYZ*, (d) *CMY*, and (e) *HSV* color spaces.

**Figure 6:**
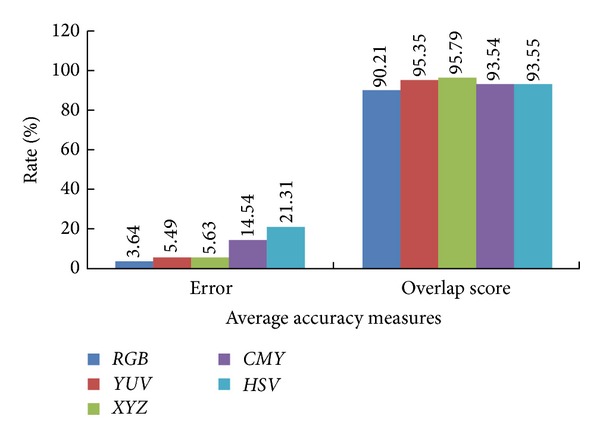
Comparison of average accuracy measures for applying automatic GrabCut segmentation on different color spaces.

In the second experiment, the automatic GrabCut, which is initialized using Orchard and Bouman, is applied with different color space models, including *RGB*, *XYZ*, *CMY*, *YUV*, and *HSV*. The features that identify each image pixel are only the values of its three components in the selected color space. The final segmentation results are obtained for all used images. For a quantitative comparison, Table 2 shows the error rate and the overlap score rate for the whole dataset. The results in Table 2 are ordered in ascending order from left to right in terms of the total number of good image segmentation results and the average error rates. We can see that the *RGB* space is the one that obtains better results for most of the images in terms of the average error rate. *YUV* and *XYZ* follow with very little increase in the average error rate. They exhibit almost the same average error and overlap score rates, which are 5.49% for the error rate and 95.35% for the overlap score rate and 5.63% for the error rate and 95.79% for the overlap score rate, respectively.  Figure 5 shows visual segmentation results for some images, while Figure 6 shows graph plots of the average segmentation error rate and the overlap score rate for all different color spaces.

## 7. Conclusions and Future Work

In this paper, a modification of GrabCut is presented to eliminate the need of initial user interaction for guiding segmentation and hence converting GrabCut into an automatic segmentation technique. The modification includes using Orchard and Bouman as an unsupervised clustering technique to initialize the GrabCut segmentation process. Based on a dataset of 23 images, the experiments revealed that automatic GrabCut using Orchard and Bouman clustering outperforms the original GrabCut. It reduces the need for user intervention while segmentation and adds extra advantage for the GrabCut via automation. Furthermore, it provides robust and accurate segmentation with average error rates of 3.64% compared to the results of 4.28% average error rate that is achieved by the original GrabCut. In addition, the performance of the automatic GrabCut is evaluated using five different color spaces, *RGB*, *YUV*, *XYZ*, *HSV*, and *CMY*. The experimental results show that the segmentation results depending on the *RGB* color space provided the best segmentation results compared to other color spaces for the considered set of images.

This study can be improved by enlarging the dataset and including different kinds of images. On the other hand, future work might include modifying the energy minimization procedure of the automatic GrabCut to allow for multilabel optimization and segmentation.

## Supplementary Material

The supplementary material includes a table showing the experimental results of a comparative study that was implemented in a previous research by the authors and which is currently submitted to the Journal of Computer Science and Technology (JCST) 2014 and under review. This study applies the automatic GrabCut which is initialized using different unsupervised clustering techniques and compares their performance based on the accuracy achieved to the problem of color image segmentation.
The study justifies the selection of the Orchard and Bouman clustering technique for the GrabCut initialization. According to the comparative study, the Orchard and Bouman clustering outperformed other unsupervised clustering techniques including Self Organizing Maps (SOFM) and Fuzzy C-means (FCM) for the automation of the GrabCut in terms of improving the segmentation accuracy and achieving the best error rate applied to the selected dataset of images.

## Figures and Tables

**Table 1:** Comparisons between the original and automatic GrabCut.

Image	Error rate %		Overlap score rate %	
	Original semiautomatic GrabCut	Automatic GrabCut using Orchard and Bouman	Original semiautomatic GrabCut	Automatic GrabCut using Orchard and Bouman
1	3.05	18.70	95.91	58.57
2	15.48	5.74	43.96	75.69
3	4.79	7.31	91.51	85.48
4	3.07	3.09	97.06	97.02
5	4.16	3.75	82.42	85.18
6	0.86	0.86	97.17	97.17
7	2.40	2.40	69.00	69.01
8	0.87	1.08	92.76	90.81
9	25.81	2.17	67.66	97.31
10	2.82	2.81	96.32	96.35
11	2.05	2.05	97.04	97.04
12	4.99	4.93	88.45	89.32
13	2.28	2.30	95.71	95.64
14	2.36	2.56	96.85	96.41
15	2.78	3.50	94.14	91.98
16	3.16	3.02	93.38	93.80
17	2.08	2.10	95.19	95.11
18	3.88	3.86	90.95	91.06
19	2.88	2.92	93.44	93.30
20	1.43	1.44	96.47	96.43
21	1.27	1.27	94.62	94.64
22	3.30	3.16	93.37	93.73
23	2.57	2.58	93.75	93.74

Avg.	4.28	3.64	89.44	90.21

SD	5.50	3.61	12.76	9.87

**Table 2:** Experimental segmentation results on different color spaces using automatic GrabCut.

Image	Error rate %					Overlap score rate %				
	*RGB *	*YUV *	*XYZ *	*CMY *	*HSV *	*RGB *	*YUV *	*XYZ *	*CMY *	*HSV *
1	**18.70**	**20.09**	5.45	**36.31**	5.21	58.57	92.99	98.47	98.05	99.24
2	**5.74**	2.79	2.92	2.91	**19.19**	75.69	98.85	98.63	97.95	98.86
3	**7.31**	**5.51**	3.90	3.76	**5.37**	85.48	95.38	99.21	99.31	98.22
4	3.09	3.07	**18.95**	3.08	**12.67**	97.02	99.19	88.13	99.11	100
5	3.75	3.76	**4.20**	**42.25**	**74.38**	85.18	89.33	85.70	99.19	96.56
6	0.86	0.86	0.89	**30.82**	**29.90**	97.17	99.51	99.56	74.05	98.20
7	2.40	2.33	2.28	1.20	**36.11**	69.01	99.85	99.76	93.21	98.43
8	1.08	**6.94**	1.08	2.68	2.68	90.81	44.91	97.27	88.69	88.74
9	2.17	2.16	2.17	**31.43**	**28.80**	97.31	99.22	99.24	82.93	85.93
10	2.81	**4.19**	**4.23**	**4.81**	**5.69**	96.35	99.90	99.92	97.39	100
11	2.05	2.07	2.18	**14.99**	**15.93**	97.04	99.91	99.59	63.79	61.16
12	4.93	**5.22**	4.97	4.44	**8.29**	89.32	92.48	93.30	96.81	81.42
13	2.30	2.39	2.42	**42.31**	**27.58**	95.64	98.73	99.03	99.91	97.09
14	2.56	2.81	3.06	**4.15**	**4.28**	96.41	98.37	97.97	94.79	94.55
15	3.50	3.68	3.68	**5.27**	**12.71**	91.98	99.28	99.28	99.22	88.40
16	3.02	2.94	2.98	**8.78**	**49.66**	93.80	98.89	98.94	99.56	99.79
17	2.10	2.20	2.12	**35.89**	**6.09**	95.11	99.07	99.00	99.65	81.17
18	3.86	**6.71**	**6.76**	**35.85**	**80.23**	91.06	98.95	98.71	96.36	100
19	2.92	**37.29**	**45.78**	**5.06**	**6.40**	93.30	99.88	64.12	90.29	99.84
20	1.44	1.43	1.47	**8.48**	**27.59**	96.43	98.95	99.10	98.04	98.82
21	1.27	1.27	1.04	1.55	**22.74**	94.64	99.70	98.99	98.18	99.92
22	3.16	3.39	3.39	**5.21**	**5.29**	93.73	94.65	94.36	89.97	89.85
23	2.58	3.21	3.56	3.25	3.28	93.74	95.13	94.91	94.95	95.58

Avg.	3.64	5.49	5.63	14.54	21.31	90.21	95.35	95.79	93.54	93.55

SD	3.61	7.92	9.47	15.24	21.64	9.87	11.37	7.84	9.01	9.29
