# Supplementary material for: Color Image Segmentation Based on Different Color Space Models Using Automatic GrabCut
Source: ScientificWorldJournal. 2014 Aug 31;2014:126025. doi: 10.1155/2014/126025 (PMC4165205; doi:10.1155/2014/126025)
Supplement: Supplementary file 1 — The supplementary material includes a table showing the experimental results of a comparative study that was implemented in a previous research by the authors and which is currently submitted to the Journal of Computer Science and Technology (JCST) 2014 and under review. This study applies the automatic GrabCut which is initialized using different unsupervised clustering techniques and compares their performance based on the accuracy achieved to the problem of color image segmentation. The study justifies the selection of the Orchard and Bouman clustering technique for the GrabCut initialization. According to the comparative study, the Orchard and Bouman clustering outperformed other unsupervised clustering techniques including Self Organizing Maps (SOFM) and Fuzzy C-means (FCM) for the automation of the GrabCut in terms of improving the segmentation accuracy and achieving the best error rate applied to the selected dataset of images. [file 126025.f1.pdf]

Table 1. Experimental segmentation results of automatic GrabCut initialized using different clustering techniques.

**Image**	**Error rate %**			**Overlap Score rate %**		
	**Orchard and Bouman**	**SOFM**	**FCM**	**Orchard and Bouman**	**SOFM**	**FCM**
1	18.70	3.15	24.92	58.57	95.68	43.97
2	5.74	2.80	2.80	75.69	93.70	93.70
3	7.31	6.00	4.95	85.48	89.12	91.21
4	3.09	3.67	61.00	97.02	95.63	38.40
5	3.75	36.48	36.54	85.18	29.16	29.13
6	0.86	0.86	0.86	97.17	97.16	97.16
7	2.40	2.40	2.40	69.01	68.93	69.00
8	1.08	2.18	1.92	90.81	80.83	82.98
9	2.17	12.87	2.15	97.31	81.31	97.35
10	2.81	4.15	4.15	96.35	94.33	94.26
11	2.05	2.17	2.17	97.04	96.71	96.71
12	4.93	4.93	4.92	89.32	89.32	89.35
13	2.30	2.30	2.33	95.64	95.64	95.53
14	2.56	3.96	2.49	96.41	93.27	96.59
15	3.50	2.89	2.89	91.98	94.03	94.05
16	3.02	2.98	2.98	93.80	94.26	94.26
17	2.10	2.16	2.16	95.11	94.75	94.75
18	3.86	3.88	3.88	91.06	90.98	90.98
19	2.92	2.88	37.10	93.30	93.43	38.35
20	1.44	1.46	1.47	96.43	96.34	96.33
21	1.27	1.27	1.28	94.64	94.64	94.61
22	3.16	3.44	3.17	93.73	93.07	93.71
23	2.58	3.27	2.56	93.74	91.15	93.78
**Average**	3.64	4.88	9.18	90.21	88.85	82.80
**SD**	3.61	7.28	15.49	9.87	14.54	22.24
